# Differential Capacity of Human Skin Dendritic Cells to Polarize CD4^+^T Cells into IL-17, IL-21 and IL-22 Producing Cells

**DOI:** 10.1371/journal.pone.0045680

**Published:** 2012-11-30

**Authors:** Karine Penel-Sotirakis, Elise Simonazzi, Josette Péguet-Navarro, Aurore Rozières

**Affiliations:** Université de Lyon, Hôpital E. Herriot, Lyon, France; University Hospital Freiburg, Germany

## Abstract

Accumulating evidence suggests a contribution of T cell-derived IL-17, IL-21 and IL-22 cytokines in skin immune homeostasis as well as inflammatory disorders. Here, we analyzed whether the cytokine-producing T lymphocytes could be induced by the different subsets of human skin dendritic cells (DCs), i.e., epidermal Langerhans cells (LCs), dermal CD1c^+^CD14^−^ and CD14^+^ DCs (DDCs). DCs were purified following a 2-day migration from separated epidermal and dermal sheets and co-cultured with allogeneic T cells before cytokine secretion was explored. Results showed that no skin DCs could induce substantial IL-17 production by naïve CD4^+^ or CD8^+^T lymphocytes whereas all of them could induce IL-17 production by memory T cells. In contrast, LCs and CD1c^+^CD14^−^DDCs were able to differentiate naïve CD4^+^T lymphocytes into IL-22 and IL-21-secreting cells, LCs being the most efficient in this process. Intracellular cytokine staining showed that the majority of IL-21 or IL-22 secreting CD4^+^T lymphocytes did not co-synthesized IFN-γ, IL-4 or IL-17. IL-21 and IL-22 production were dependent on the B7/CD28 co-stimulatory pathway and ICOS-L expression on skin LCs significantly reduced IL-21 level. Finally, we found that TGF-β strongly down-regulates both IL-21 and IL-22 secretion by allogeneic CD4^+^ T cells. These results add new knowledge on the functional specialization of human skin DCs and might suggest new targets in the treatment of inflammatory skin disorders.

## Introduction

The skin represents a crucial barrier against the external environment, any breach of which requires a vigorous immune response. Dendritic cells (DCs) are specialized antigen-presenting cells with the unique ability to initiate primary T cell immune responses [1], [2], [3]. Within human skin, different types of myeloid DCs are found in the two distinct epidermal and dermal layers [4]. Langerhans cells (LCs) are located in the basal and supra basal layer of the epidermis whereas the dermis was recently shown to contain at least two migratory CD1a^+^CD14^−^ and CD1a^+/−^CD14^+^DC subsets [5], [6].

In response to danger signals leading to the local production of pro-inflammatory cytokines, DCs undergo a complex process of activation/maturation allowing their migration to draining lymph nodes where they initiate naïve T cell proliferation and differentiation [1], [4]. Especially, DCs acquire or up-regulate a unique set of cell-surface proteins which bind to specific receptors on lymphocytes and regulate naïve T cell activation. Thus, the CD80 or CD86 interaction with CD28 is essential for T cell activation and both Th1 and Th2 cytokine secretion. Several newly characterized members of the B7 family such as inducible co-stimulator ligand (ICOS-L/B7-H2/CD275) and programmed death ligand-1 (PD-L1, B7-H1/CD274) also have critical role in controlling immune responses [7], [8], [9].

Depending on many factors, including their activation/maturation state and the cytokines they produce, DCs really control the differentiation of naïve CD4^+^T cells into different helper subsets. These subsets exhibit distinct cytokine profile and effector function, thereby providing protection from various types of pathogens. In addition to the classical Th1 (producing IL-2 and IFN-γ) and Th2 (IL-4, IL-5, IL-13), Th17 have been later classified as a separate lineage of helper T cells able to produce IL-17A and IL-17F, as well as IL-21 and IL-22 [10]. Th17 cytokines act mainly at outer body barriers such as skin and their primary function is the clearance of pathogens that require a massive inflammatory response. Conversely, these cytokines have been implicated in autoimmune diseases as well as the pathogenesis of many inflammatory skin disorders such as atopic dermatitis and psoriasis. Both IL-17A and F induce multiple proinflammatory mediators, including chemokines and cytokines, from epithelial and fibroblast cells [11], thereby promoting attraction of neutrophils to the site of inflammation.

In contrast to other T cell cytokines, IL-22 does not act on immune cells but mostly targets epithelial cells. Within human skin, IL-22 acts in cooperation with IL-17 to induce anti-microbial peptide activation in keratinocytes [12], [13] thereby enhancing the clearance of bacterial infections. IL-22 also mediates keratinocyte proliferation and epidermal hyperplasia and is thought to play a central role in wound healing as well as in inflammatory diseases such as psoriasis [14]. Although IL-22 was initially identified as a Th17 cytokine, there is increasing evidence that a “Th22” cell subset can produce IL-22 without IL-17 and IFN-γ [15], [16], [17]. In particular, Th22 cells were found among the resident T cells in normal human skin [18].

IL-21, originally classified as a Th2 cytokine, was strongly associated with T follicular helper cells (Tfh), capable of providing help for B cell differentiation and isotype switch [19]. However, Th17 were reported as the strongest producers of IL-21 which, in turn, further amplifies Th17 generation in an autocrine manner [10]. Within human skin, IL-21 promotes keratinocyte proliferation and is thought to be involved in the epidermal hyperplasia of psoriasis [20].

Although Th17 cytokines have been clearly detected within human skin, the capacity of skin DCs to initiate the cytokine production has been little studied. Indeed, Klechevsky et al. first [6] reported a functional specialization of human skin DCs, LCs being the most efficient at priming both Th2 and cytotoxic CD8^+^T cell response and CD14^+^ dermal DCs (DDCs) the only skin DCs able to polarize Th-like cells. Recently, we showed that human migratory LCs were better inducers of both Th1 and Th2 naïve CD4^+^T cell differentiation than CD14^−^CD1c^+^DDCs, a process that involves distinct co-stimulatory activity [21].

In the present study, we compared the capacity of human epidermal LC, CD1c^+^CD14^−^ and CD14^+^ DDCs, migrating from the same skin explants, to drive IL-17, IL-22 and IL-21 cytokine production by allogeneic naïve T cells.

## Methods

### Purification of human skin DCs following migration from epidermal and dermal sheets

Normal human skin was obtained from healthy donors undergoing abdominal plastic surgery, as previously described [21]. Samples were recovered after informed written consent and used according to the guidelines of the Declaration of Helsinki Principles. The study was approved by the institutional review board of the University Claude Bernard, Lyon, France.

Briefly, 3-mm-thick skin slices were incubated in Hanks' Balanced Saline Solution (HBSS) containing 0.25% dispase II (Roche Diagnostics, Meylan, France) and antibiotics, for 1 hr at 37°C. Epidermal and dermal sheets were separated using fine pliers and washed with HBSS. Dermal sheets were carefully scratched with curved pliers to remove any residual epidermal cells. The sheets were then placed in separate Petri dishes containing X-vivo-15 medium (Cambrex, East Rutherford, U.S.A.) supplemented with antibiotics and cultured for 48 hrs at 37°C in a humidified atmosphere. Migratory cells were harvested, filtered onto gauze, washed in HBSS and counted. LCs were purified by successive centrifugations on Lymphoprep (Flobio SA, Courbevoie, France) and Nycoprep, respectively [21]. Dermal migratory CD14^+^cells were purified using CD14 microbeads according to the manufacturer's protocol (Miltenyi Biotec, Bergisch Gladbach, Germany). Remaining CD14^−^ cells were further purified using human anti-CD1c-biotin coupled to anti-biotin microbeads (Miltenyi Biotec). DC purification was checked by cell surface staining followed by flow cytometry analysis. The purity of LC and DDC suspensions was routinely more than 90% [21]. Viability of purified DC suspensions routinely exceeded 98%, as assessed by trypan blue exclusion test. Cells were then used for mixed lymphocyte reaction (MLR).

### Phenotypic analysis

Migratory LCs and DDCs were pre-incubated with human AB serum to block Fc receptors. Cells were incubated for 30 minutes at 4°C and controls were carried out with irrelevant isotype matched Igs. The following mAbs were used: anti-HLA-DR (B8.12.2); anti-CD1a (NA1/34) from Dako (Glostrup, Denmark); anti-CD1c (AD5- 8E7) from Miltenyi Biotech; anti-CD80 (MAB 104), and anti-CD83 (HB15A), all from Immunotech (Marseille, France); anti-CD86 (2331FUN-1) from BD Pharmingen (San Diego, CA); anti-PD-L1/B7-H1 (MIH1), ICOS-L, B7/H2 (MIH12)(from eBiosciences, San Diego, CA).

### T cell purification

Normal allogeneic naïve CD4^+^ or CD8^+^T cells were purified from human umbilical cord blood. Mononuclear cells were obtained by density centrifugation on Lymphoprep followed by positive magnetic-selection, using CD4 or CD8-coupled microbeads according to the manufacturer's protocol (Miltenyi Biotec). Both CD4^+^ and CD8^+^T cell populations were routinely 96% to 98% pure and contained more than 95% CD4^+^ or CD8^+^/CD45RA^+^ naïve T cells, as assessed by flow cytometry using a FACScan together with the CELLQuest Software (Becton Dickinson, Le Pont de Claix, France).

In some experiments, total allogeneic T cells were purified from the peripheral blood of healthy donors, as previously described [21]. Total CD4^+^T cells were isolated by positive magnetic selection, using the CD4 microbeads according to the manufacturer's protocol (Miltenyi Biotec).

Each sample was recovered after informed written consent and used according to the guidelines of the Declaration of Helsinki Principles. The study was approved by the institutional review board of the University Claude Bernard, Lyon, France.

### T cell proliferation assays

Mixed LC or DDC-lymphocyte reactions were carried out in 96-well round-bottomed microtiter plates. Purified migratory LC, CD1c^+^CD14^−^DDCs or CD14^+^DDCs were added in serial two-fold dilutions to 10^5^ allogeneic naïve CD4^+^T cells. Controls with DCs or T cells alone were included in each experiment. Culture medium was RPMI-1640 (Gibco Laboratories, Grand Island, NY) supplemented with 10% human AB serum and antibiotics. Triplicate cultures were maintained for 5 days at 37°C in a 5% CO_2_ humidified atmosphere. T cell proliferation was measured by pulsing the cells with 1 µCi of [^3^H]methylthymidine (5 Ci/mmol; Amersham Pharmacia Biotech, Les Ulis, France) for the final 18 hours of culture. Cells were then harvested, and incorporated thymidine was quantified in a direct beta counter (Matrix 96; Packard, Downers Grove, IL). Results were expressed as the mean counts per minute±SD of triplicate cultures.

### Polarization of allogeneic T cells

Purified migratory DCs were added to allogeneic naïve CD4^+^T cells at 1∶10 APC/T cell ratio in flat-bottomed microtiter plates for 6 days at 37°C in a 5% CO_2_ humidified atmosphere. Culture medium was RPMI-1640 medium supplemented with 10% human AB serum or, when mentioned, serum-free X-vivo-15 medium, and antibiotics. When indicated, naïve T cells were replaced by total or CD4^+^ allogeneic T cells, both containing memory T cells.

In some experiments, DCs were pre-incubated for 30 minutes at 4°C with anti-human PDL-1/B7-H1/CD274 (40 µg/ml), ICOS-L/B7-H2/B7RP-1/CD275 (40 µg/ml; both from eBiosciences, San Diego, CA), or recombinant CTLA-4/Fc chimera (1 µg/ml, R&D Systems Europe, Abingdon, England) with the ability to bind both CD80 and CD86, before adding the naïve CD4^+^T cells. Alternatively, mixed leukocyte reactions (MLR) were carried out in the presence of anti-human TGF-β (10 µg/ml, R&D Systems) or exogenous recombinant human TGF-β (50 ng/ml, R&D Systems). Control Igs were used as control.

For enzyme-linked immune-absorbent assay (ELISA), cells were re-stimulated after 6 days by phorbol myristate acetate (PMA) (50 ng/ml) and ionomycin (1 µg/ml) both from Sigma-Chemicals (St Louis, MO). DCs or T cells alone were used as control. Supernatants were collected 24 hours later and stored at −80°C until cytokines were measured.

For intracellular cytokine staining, cells were treated with PMA and ionomycin for 6 hours only and blockage of cytokine secretion was achieved by simultaneous treatment with brefeldin A (10 µg/ml, Sigma) for the last 5 hours.

### ELISA

IL-6 and TNF-α were assayed using ELISA methods (Human IL-6 Duoset Elisa and Human IL-6 Duoset Elisa Rand D system Minneappolis, USA in supernatant after 48 hours of DC stimulation with or without LPS (100 ng/ml) in complete medium.

IL-21 and IL-22 levels were assayed using ELISA Max™ Deluxe sets from Biolegend (San Diego, CA). IL-17 levels were analyzed using the IL-17A ELISA kit from Gen-probe Lifesciences Ltd (Manchester, UK).

### Intracellular cytokine staining

Human CD4^+^T lymphocyte were fixed and permeabilized with the Inside Stained Kit (Miltenyi Biotec). Intracellular cytokine staining was performed using anti-IFN-γ-Alexa Fluor 488, anti-IL17-Alexa Fluor 488, anti-IL-4-phycoerythin (PE) (all from Becton Dickinson, Le Pont de Claix, France), anti-IL-17-PE (R&D systems, Lille, France), anti-IL4-Alexa Fluor 488, anti-IL-21-PE and anti-IL-22-PE (eBioscience). In some experiments, membranous cutaneous lymphocyte-associated antigen (CLA) was detected using FITC-conjugated anti-CLA mAb (Becton Dickinson). Appropriate fluorochrome-conjugated irrelevant isotype-matched mAbs were used as controls. Proliferating T lymphocytes were gated according to SSC/FSC parameters and florescence was analyzed by flow cytometry.

### Statistical analysis

Results were analyzed for statistical significance using the Wilcoxon matched pairs signed-rank test. Only p values <0.05 were considered to be significant.

## Results

### 1- Migratory LCs display higher allostimulatory capacity than migratory CD1c^+^CD14^−^ and CD14^+^DDCs

**Figure 1:**
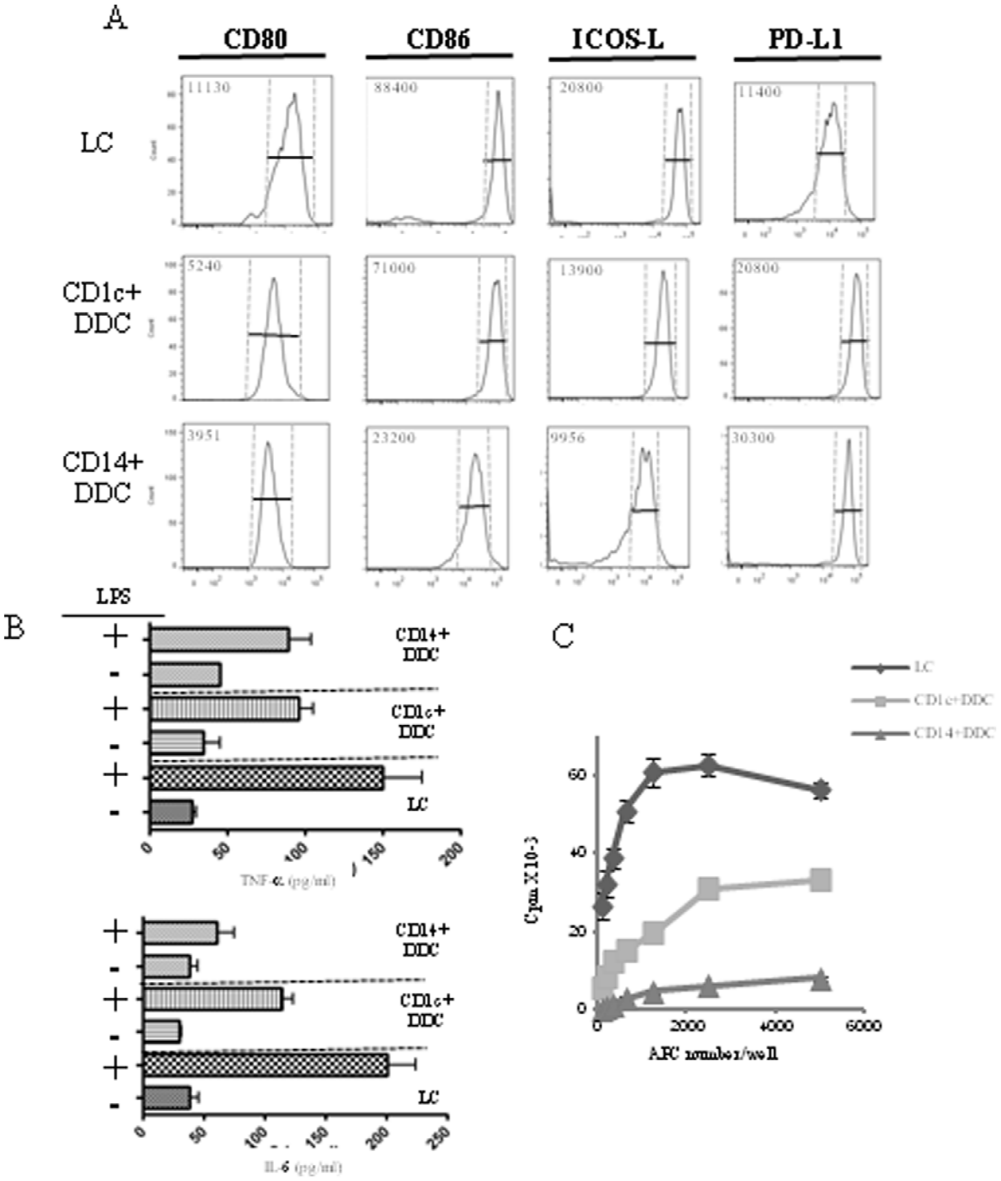
Langerhans cells are stronger stimulators of allogeneic naïve CD4^+^T cell proliferation than dermal DCs. A. Phenotypic analysis of co-stimulatory molecules on migratory skin DC. CD80, CD86, ICOS-L and PD-L1 expression were measured by Flow Cytometry method B. Il-6 and TNF-α secretion after LPS stimulation of migratory skin DC. Migratory skin DC were stimulated with LPS. After 48 hours of stimulation IL-6 and TNF-α were measured by ELISA methods. Results are express in pg/ml ±SD of triplicate cultures and representative of three experiments C Allogeneic proliferation capacity. Purified migratory human skin DCs were added in graded numbers to allogeneic naïve CD4^+^T cells. Proliferation was measured after 5 days by ^3^H-thymidine incorporation. Results are the mean counts per minute (cpm) ±SD of triplicate cultures and representative of three experiments. T cells alone gave less than 100 cpm.

Skin DCs were obtained after enzymatic splitting of the epidermis and dermis and subsequent culture in serum free medium allowing spontaneous DC migration. In such conditions, we demonstrated that migratory LCs displayed a more activated phenotype than migratory DDCs, with a stronger expression of CD80 and CD86 co-stimulatory molecules (Figure 1A) [6], [21] and associated with a higher expression of ICOS-L and a lower expression of PD-L1 (molecules from B7 family) in LCs. The ability to induce proliferation of allogeneic naïve T cells is one of the definitive features of DCs. As depicted in Figure 1C, LCs appeared superior to CD1c^+^CD14^−^ and especially CD14^+^DDCs at inducing the proliferation of naïve CD4^+^T cells. The allogeneic capacity of LCs correlated with a higher ability to produce Th polarizing cytokines as (IL-6 and TNF-α) after DC stimulation (Figure 1B)

### 2- No skin DCs can induce IL-17 production by naïve T lymphocytes

We analyzed the relative capacity of human skin migratory DCs to trigger IL-17 production by naïve T lymphocytes. To this end, a 6-day mixed leukocyte reaction (MLR) was performed and the cells were further stimulated with polyclonal T lymphocyte stimuli.

**Figure 2:**
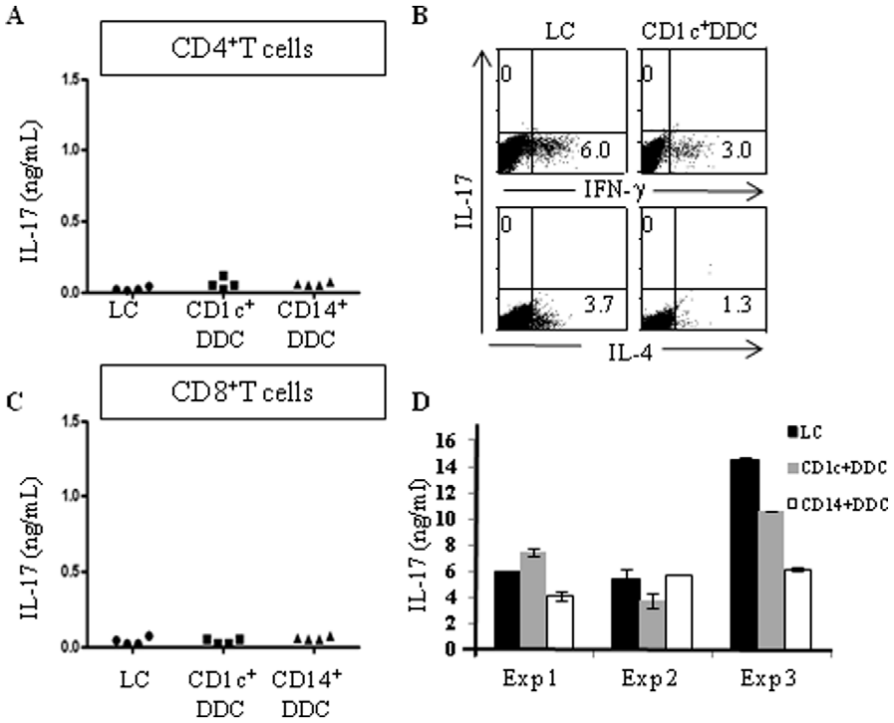
No skin dendritic cells (DCs) can induce IL-17 production by naïve T cells. Purified migratory skin DCs were cultured with allogeneic CD4^+^ (A, B), CD8^+^ (C) cord blood naïve T cells or adult peripheral blood total T cells (D) for 6 days and restimulated with phorbol myristate acetate (PMA) and ionomycin. (A, C, D) Cell supernatants were harvested after 24 hours and IL-17 secretion was measured by ELISA. Results are from six (A), four (C) or three duplicate (D) experiments carried out with different donors. (B) Intracytoplasmic cytokines were analyzed after 6 hours and results are representative for 7 experiments carried out with different donors.

As assessed by ELISA, neither of human skin DCs was able to induce naïve CD4^+^T lymphocytes to secrete substantial IL-17 levels. Thus, supernatants from naïve CD4^+^T lymphocytes stimulated by LCs, CD1c^+^CD14^−^ or CD14^+^ DDCs contained very low or even no detectable IL-17 levels (16±14; 49±56 and 14±10 pg/ml, respectively, n = 6; Figure 2A). Human AB serum has been reported to inhibit Th17 differentiation [22]. However, similar results were obtained when MLR were carried out using X-vivo-15 serum free medium (data not shown).

These data were confirmed by intracellular cytokine staining (Figure 2B). As reported earlier [21], CD1c^+^CD14^−^DDCs and especially LC were very efficient at inducing IFN-γ and IL-4 production by naïve CD4^+^T cells. In these conditions, no IL-17 producing T lymphocytes could be detected (Figure 2B).

Furthermore, neither of human skin DCs was able to induce allogeneic naïve CD8^+^ T lymphocytes to secrete IL-17 (Figure 2C).

Interestingly, however, each DC types could induce high IL-17 production when co-cultured with total peripheral blood T cells, mainly comprising memory T lymphocytes (Figure 2D). Similar results were obtained when DCs were co-cultured with total peripheral blood CD4^+^T lymphocytes (data not shown).

### 3- Migratory LCs are the most efficient skin DCs at polarizing Th22 cell subset

**Figure 3:**
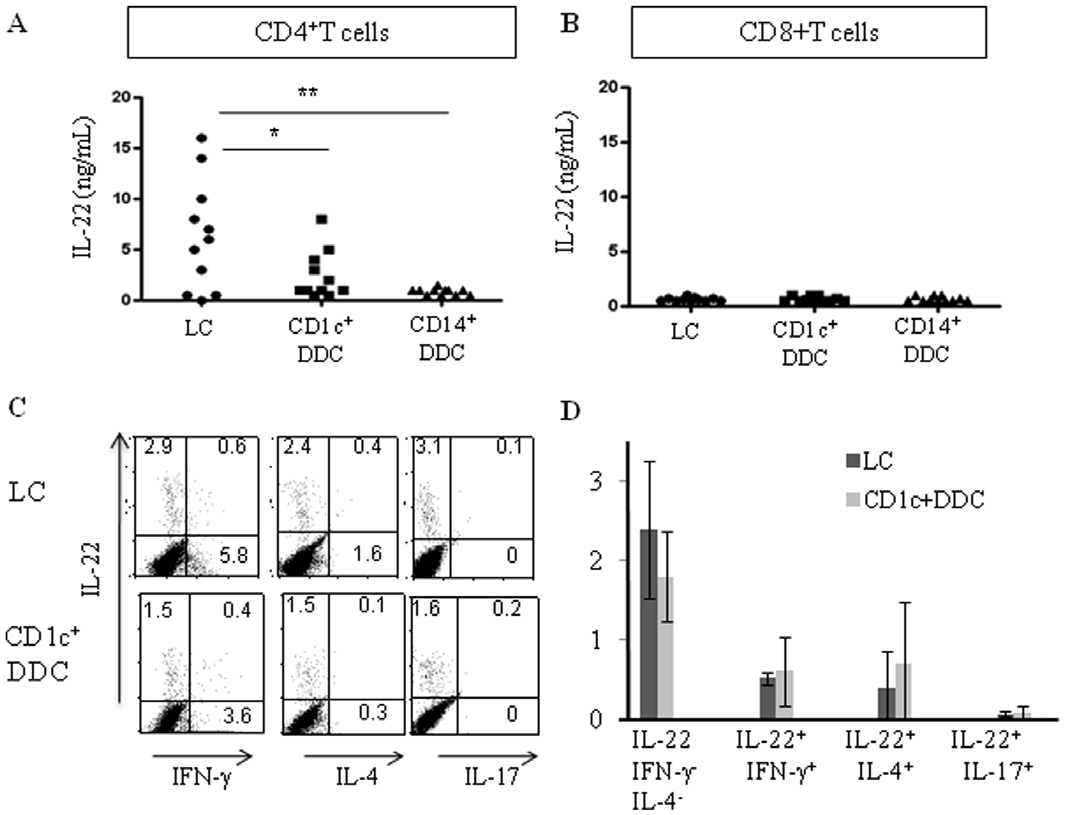
Migratory Langerhans cells (LCs) are the most efficient skin dendritic cells (DCs) at polarizing Th22 cell subset. Purified migratory skin DCs were cultured with allogeneic CD4^+^ (A, C, D) or CD8^+^ (B) naïve T cells for 6 days and restimulated with phorbol myristate acetate (PMA) and ionomycin. (A, B) Cell supernatants were harvested after 24 hours and IL-22 secretion was measured by ELISA. Each marker illustrates experiments performed with different donors. (A) ** p<0.001, *p<0.002, as compared with LC values. (C, D) Intracytoplasmic cytokines were analyzed after 6 hours. (D) Results are the mean±SD percentage of stained cells from 4 experiments carried out with different donors.

MLR supernatants from skin DC-stimulated naïve CD4^+^T cells were then assayed for IL-22 production. As illustrated in Figure 3A, LCs induced higher production of IL-22 cytokine than CD1c^+^CD14^−^DDCs (6787±4600 versus 2415±2260 pg/ml, respectively, p<0.002, n = 11). In contrast, CD14^+^DDC-stimulated CD4^+^T lymphocytes secreted very low levels of IL-22, if any (65±125 pg/ml). In these experiments, supernatants from purified DCs or control T lymphocytes cultured alone did not contain detectable IL-22 levels (data not shown). Moreover, very low levels of IL-22 were found in LPS-, poly(I:C)- or CD40L and IFN-γ-activated skin DCs (64±15 pg/ml for LC, n = 5, data not shown).

In contrast to naïve CD4^+^T cells, naïve CD8^+^T lymphocytes from the same donors were unable to secrete detectable amount of IL-22 (Figure 3B).

We next examined whether IL-22 producing naïve T cells co-produced Th1, Th2 or Th17 cytokines, using double intracellular cytokine staining followed by flow cytometry analysis. CD1c^+^CD14^−^DDCs and especially LCs induced consistent frequencies of IL-22-producing cells (Figures 3C and 3D). As expected, these cells did not co-produce IL-17. Moreover, only a limited proportion co-synthesized IFN-γ or IL-4. Thus, most of the IL-22^+^ T lymphocytes belong to the Th22 subset.

### 4- Migratory LCs are the most potent skin DCs at inducing naïve CD4^+^T lymphocytes to produce IL-21

**Figure 4:**
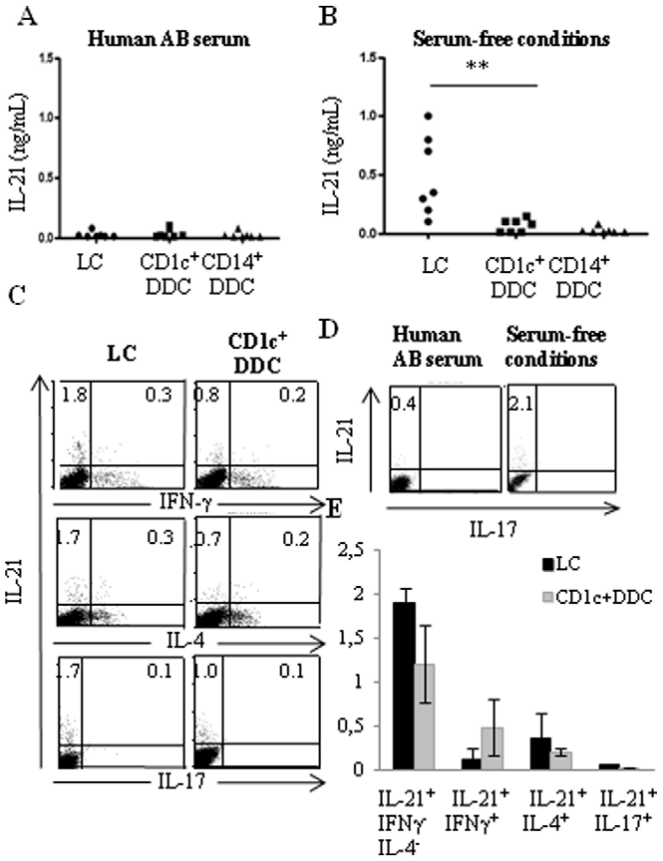
Migratory Langerhans cells can induce IL-21 production by naïve CD4^+^T cells in serum-free conditions. Purified migratory skin DCs were cultured with allogeneic CD4^+^ naïve T cells for 6 days and restimulated with phorbol myristate acetate (PMA) and ionomycin. Culture medium was (A) RPMI-1640 supplemented with 10% human AB serum or (B, C, E, F) X-vivo 15 serum free medium. (A, B) Cell supernatants were harvested after 24 hours and IL-21 secretion was measured by ELISA. Results were from (A) five or (B) seven experiments performed with different donors. (C, D, E, F) Intracytoplasmic cytokines were analyzed after 6 hours. (D) Results are from the same experiment carried out in either serum-supplemented or depleted medium.

Very low levels of IL-21 were found in supernatants from MLR carried out in serum-supplemented medium (53 (pg/ml) ±26, 39±18 and 25±12 for T cells stimulated with LCs, CD1c^+^CD14^−^ or CD14^+^DDCs, respectively, n = 7, Figure 4A). Surprisingly, IL-21 production was significantly up-regulated in serum-free cultures (Figures 4B and 4D), suggesting that the serum contained inhibitory factors. Migratory LCs appeared far more potent than CD1c^+^CD14^−^DDCs at inducing IL-21 production whereas CD14^+^DDCs induced barely detectable levels (Figure 4B). Importantly, supernatants from naïve CD4^+^T cells cultured alone or from purified skin DCs stimulated or not with CD40L-tranfected fibroblasts and IFN-γ did not contain detectable levels of IL-21.

Unlike naïve CD4^+^T lymphocytes, naïve CD8^+^T lymphocytes were unable to secrete IL-21 (data not shown).

As shown by intracellular cytokine staining, T lymphocytes stimulated by LCs or, to a lower extent, CD1c^+^CD14^−^DDCs induced significant frequencies of IL-21-producing cells (Figure 4C and 4F). As expected, the cells did not stain with anti-IL-17 mAb. Only a very limited proportion of them co-produced IFN-γ or IL-4 (Figure 4C and 4F). Therefore, the largest subset of IL-21^+^ T lymphocytes appeared to represent a unique subset of IL-21-producing helper T lymphocytes (Figure 4E).

### 5- ICOS-L expression on LCs down-regulates IL-21 production by naïve CD4^+^T cells

**Figure 5:**
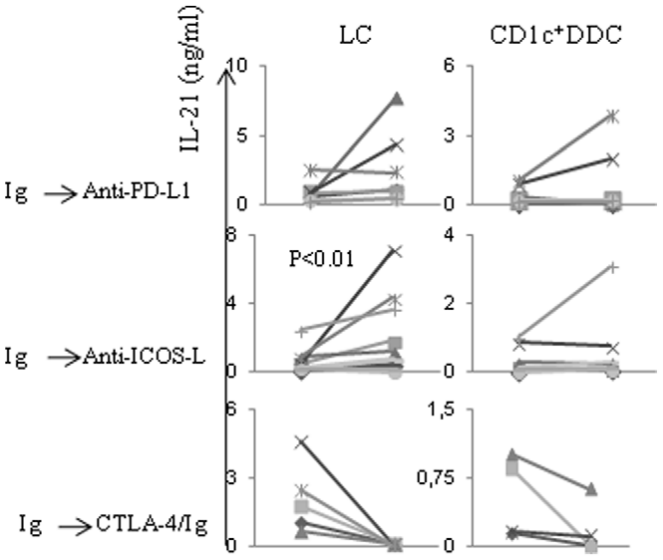
ICOS-L expression on Langerhans cells down-regulates IL-21 production by naïve CD4^+^T cells. Purified migratory skin DCs were treated with anti-PD-L1, anti ICOS-L mAbs (40 µg/ml), CTLA-4/Ig fusion protein (1 µg/ml) or control Ig before being added to allogeneic CD4^+^ naïve T cells. After a 6 day co-culture in serum-free medium, cells were restimulated with phorbol myristate acetate (PMA) and ionomycin. Cell supernatants were harvested 24 hours later and IL-21 secretion was measured by ELISA. Each marker illustrates experiments carried out with different donors. Statistical analysis was performed between control and test groups and only significant difference was indicated.

Molecules of the B7 family are well-known to regulate Th1 or Th2 cytokine production by T cells. We showed that migratory CD1c^+^CD14^−^ and CD14^+^ DDCs exhibit higher PD-L1 and lower ICOS-L expression as compared to LCs (Figure 1A) and that PD-L1 limited the capacity of the DDCs to induce IFN-γ production by naïve CD4^+^T cells [21]. Here, we failed to demonstrate any reproducible effect of either anti-PD-L1 or ICOS-L mAb on IL-22 production (Figure S1). In contrast, the LCs-induced IL-21 production tended to increase in the presence of anti-PD-L1 mAb (Figure 5). Most importantly, it was significantly increased in the presence of anti-ICOS-L mAb (p<0.01), demonstrating that the molecule down-regulates IL-21 production by naïve CD4^+^T cells. CD1c^+^CD14^−^DDCs only induced low levels of IL-21 and addition of anti-PD-L1 or ICOS-L (40 µg/ml) did not significantly alter the results (Figure 5), suggesting that LCs and DDCs differently regulate IL-21 production.

Finally, the B7/CD28 co-stimulatory pathway was involved in both IL-21 and IL-22 production, as evidenced by substantial inhibition of the cytokine production in the presence of CTLA-4-Ig, at a concentration as low as 1 µg/ml (Figure 5 and Figure S1).

### 6- TGF-β down-regulates IL-22 production by naïve CD4^+^T cells

TGF-β is largely produced in skin inflammatory conditions and represents a critical cytokine for Th17 differentiation [10]. Here, we found that addition of TGF-β to the skin DC-stimulated naïve T cells, at concentration as high as 50 ng/ml, did not trigger detectable IL-17 production (data not shown).

**Figure 6:**
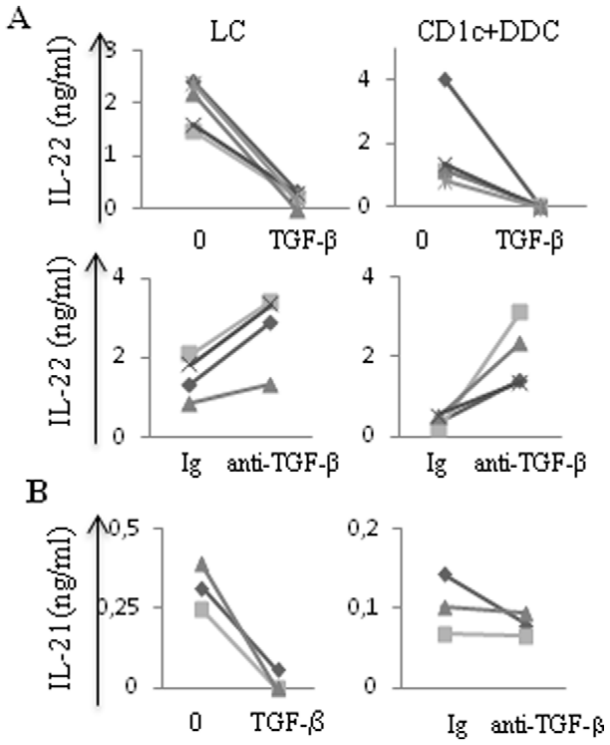
TGF-β down-regulates both IL-22 and IL-21 production by naïve CD4^+^T cells. Purified migratory skin DCs were cultured with allogeneic naïve CD4^+^T cells in the presence of exogeneous human TGF-β (50 ng/ml) or anti-human TGF-β mAb (10 µg/ml). Culture medium was RPMI 1640 supplemented with 10% human AB serum or (B, left panel) serum free medium. After 6 days cells were restimulated with phorbol myristate acetate (PMA) and ionomycin. Cell supernatants were harvested after 24 hours and (A) IL-22 or (B) IL-21 secretion was measured by ELISA. Each marker illustrates experiments carried out with different donors.

However, addition of TGF-β into the co-cultures carried out with LCs or CD1c^+^CD14^−^DDCs nearly abrogated IL-22 secretion (Figure 6A). Conversely, the blockade of TGF-β signaling consistently increased the cytokine production (Figure 6A). Moreover, in serum-free conditions, TGF-β abrogated IL-21 production by LC-stimulated naïve CD4^+^T cells (Figure 6B). As described above, IL-21 secretion was very low in serum-supplemented medium and addition of anti- TGF-β mAb did not substantially alter the results.

## Discussion

Because IL-17, IL-22 and IL-21 have been largely implied in skin homeostasis as well as inflammatory skin disorders such as psoriasis and atopic dermatitis, it is of importance to know which skin DC can induce the cytokine-producing T cells. Here, we used spontaneous migration from human epidermal and dermal sheets to recover the skin DCs. This model might be physiologically relevant since the migratory DCs exhibit a mature phenotype [21].

Only few studies have analyzed whether human cutaneous DC are able to initiate IL-17 production by naïve T cells and the results are still debated. In our experimental conditions, neither migratory epidermal LC, CD1c^+^CD14^−^DDC nor CD14^+^DCs were able to induce substantial IL-17 production by naïve T cells. This is in line with two previous studies using either CD34^+^ stem cell-derived human skin-like DC, migratory LC or CD1c^+^DDC [16], [23]. Importantly, even skin LCs stimulated with different TLR agonists [23] failed to trigger IL-17 production. Naïve T cells were shown to produce IL-17 when stimulated with Th17-related stimuli, i. e. IL-1β, IL-6, IL-23 and TGF-β [23], suggesting that some of them are missing in skin DCs, at least in these experimental conditions. In a previous study, Mathers et al. [24] found that LCs, but not DDCs, migrating from total skin explants could induce low IL-17 production by peripheral blood naïve CD4^+^T cells. The discrepancy between these and other results is unclear. It might be due to methodological factors including the protocol of LC recovery. Alternatively, some contaminating memory T cells in either naïve T cell or LC suspensions might be induced to produce IL-17 upon co-culture, thus falsifying the results. Indeed, we showed here that each skin DC subset was very strong inducers of IL-17 production by total T cells, mainly comprising memory T cells. These results extend the previous work using human CD34^+^-derived LC and DDC as APCs [23]. Aliahmadi et al. found that LC purified from human epidermal cell suspensions required TLR2 activation to induce IL-17 production by total CD4^+^ T cells [25]. This is in contrast with our results and might reflect the fully mature state of migratory, as compared to extracted, LC [6], [21]. Interestingly, the skin is stably colonized by long-lived populations of memory T cells [26]. These cells might be activated locally by skin DCs and represent an important source of IL-17, thereby controlling localized infections. In line with this, a recent paper showed that T cells isolated from normal human dermis contained as high proportion of IL-17A-producing CD4^+^T cells as psoriatic skin, indicating they are not restricted to pathological conditions [27]. At present, it is unclear whether IL-17 cytokine derived from pre-committed memory Th17 or other memory T cell subsets, however.

A distinct Th22 population has been recently characterized in the peripheral blood from healthy donors [15], [17]. This memory subset produces IL-22 in the absence of IFN-γ, IL-4 and IL-17 and possesses skin-homing properties. Very interestingly, we showed here that a substantial proportion of Th22 could be generated *in vitro* from naïve CD4^+^T cells upon stimulation with human skin DCs. LCs were more efficient than CD1c^+^CD14^−^DDCs, whereas CD14^+^DDC were unable to trigger substantial IL-22 production. This corroborates and extends the recent work by Fugita et al. [16] who analyzed the cytokine production profile of naïve T cells stimulated by LC and CD1c^+^DDCs, using intracellular cytokine staining only [16]. As compared to other skin DCs, LCs express CD1a at high level. De Jong et al. recently identified unexpectedly high frequency of CD1a-autoreactive CD4^+^ memory T cells in normal human skin [28]. Interestingly, the cells preferentially produced IL-22 in response to CD1a-bearing cells and have all the known properties of Th22 cells. This raises the question of whether CD1a might be involved in the priming of Th22 from naïve CD4^+^ T cells.

Although IL-22 was first identified as a Th17 cytokine, the relationship between IL-17 and IL-22 is much debated, especially in humans. Both cytokines contribute to the control of extracellular bacterial infection. However, increasing data now support differential regulation of IL-17 and IL-22 production during T helper cell differentiation. Thus, *in vitro* models of CD4^+^T cell priming using polarizing cytokines showed no correlation between IL-17 and IL-22 production [29]. Moreover, IL-22 did not correlate with any of the Th17 transcription factors [30]. In normal human dermis, the majority of IL-22 producing cells did not produce IL-17 [18], [27]. In line with this, we confirmed here that skin DCs can induce IL-22 production by naïve CD4^+^T cells in the absence of IL-17. Moreover, we showed that IL-22 production induced by skin DCs was down-regulated by TGF-β, whereas the cytokine is an essential component for Th17 priming [10], [22]. This is in line with a previous report using CD3/CD28-stimulated naïve T cells cultured under Th22 polarizing conditions [29]. All together, these results reinforce the concept that IL-17 and IL-22 cytokines are differently regulated during T helper cell differentiation. Future experiments must explore the regulators of Th22 development.

An interesting finding is that LCs and to a lower extent CD1c^+^DDCs were able to polarize naïve CD4^+^T lymphocytes toward IL-21 secreting lymphocytes. The majority of these cells did not belong to Th1, Th2 or Th17 subsets. To our knowledge, these are previously unreported data. Of note, the results were obtained in serum free conditions only, suggesting that the serum contains an inhibitory factor. IL-21 production was down-regulated by TGF-β which is present in human AB serum (our unpublished results). However, addition of anti-TGF-β mAb to serum-supplemented co-cultures did not result in increased IL-21 production, suggesting that TGF-β is not the only inhibitory serum factor. Further studies are needed to identify IL-21 producing cells more precisely. Interestingly, IL-21 levels were down-regulated by ICOS-L expression on LCs, providing a way for the cells to control the cytokine production.

We showed here that both IL-22 and IL-21 production depend on the B7/CD28 co-stimulatory pathway, as assessed by the strong decrease in the cytokine levels after addition of CTLA-4-Ig to the skin DC-T cell co-cultures. If the present *in vitro* results have physiological relevance, targeting of CD80 and CD86 might represent a potential way to down-regulate excessive cytokine production, notably in some inflammatory disorders. However, CTLA-4-Ig was reported to enhance the frequency of IL-17-producing CD4^+^T cells *in vitro* [31], emphasizing the importance and complexity of co-stimulatory pathways in the regulation of T cell development and the caution to be used in B7-based immunotherapy.

The role of the various skin DC subsets in the induction of effector T cell responses is still a matter of debate. Our essential knowledge derives from studies using mouse experimental models in which some reports have challenged the role of LC in skin immune responses and emphasized that of dermal DCs, notably in T cell priming against viruses or parasites [26], [27], [28]. In humans, others and our *in vitro* results highlight the essential role of LCs in the priming of CD8^+^ cytotoxic T cells [15], CD4^+^T cell differentiation into Th1, Th2 [10], [16] and, as described here, Th22 cells. This is particularly important due to the interest surrounding transcutaneous vaccination strategies. As compared to the other skin DCs, migratory LCs display far more activated phenotype and higher allostimulatory function than DDCs [10], [15], [16], which likely contribute, at least in part, to the stronger ability of the cells to induce cytokine production. Whether as yet unclear intrinsic qualities or environmental factors account for their superior capacity to differentiate naïve T cells remains to be determined. Moreover, future studies must analyze whether the present results could be reproduced in more physiological conditions using *in vitro* autologous priming to strong sensitizers for example.

In conclusion, we have shown that none of human migratory skin DCs was able to induce IL-17 production by naïve allogeneic T cells *in vitro*, whereas each of them stimulate high IL-17 production by memory T cells. In contrast, CD1c^+^CD14^−^DDC and especially LC can differentiate naïve CD4^+^T cells into IL-22 and IL-21 producing cells. This process might be an important way to directly promote the innate non-specific immunity of the tissue, thereby contributing to skin homeostasis.

## Supporting Information

Figure S1 **IL-22 production by naïve CD4^+^T cells is not regulated by PD-L1 or ICOS-L expression on skin DCs.** Purified migratory skin DCs were treated with anti-PD-L1, anti ICOS-L mAbs (40 µg/ml), cytotoxic T-lymphocyte antigen 4 (CTLA-4)/Ig fusion protein (1 µg/ml) or control Ig before being added to allogeneic CD4^+^ naïve T cells. After a 6 day co-culture, cells were restimulated with phorbol myristate acetate (PMA) and ionomycin. Cell supernatants were harvested after 24 hours and IL-22 secretion was measured by ELISA. Each marker illustrates experiments carried out with different donors.
(TIF)
